# Search for Ancient Selection Traces in Faverolle Chicken Breed (*Gallus gallus domesticus*) Based on Runs of Homozygosity Analysis

**DOI:** 10.3390/ani15101487

**Published:** 2025-05-20

**Authors:** Anna E. Ryabova, Anastasiia I. Azovtseva, Yuri S. Shcherbakov, Artem P. Dysin, Natalia V. Dementieva

**Affiliations:** Russian Research Institute of Farm Animal Genetics and Breeding (RRIFAGB)—Branch of the L.K. Ernst Federal Science Centre for Animal Husbandry, Pushkin, St. Petersburg 196601, Russia; ase4ica15@mail.ru (A.I.A.); yura.10.08.94.94@mail.ru (Y.S.S.); artemdysin@mail.ru (A.P.D.)

**Keywords:** Faverolles, whole-genome sequencing, runs of homozygosity, genome architecture, selection traces, meat quality

## Abstract

Runs of homozygosity (ROHs) are continuous homozygous segments of genomes that can be used to infer the historical development of the population. ROH studies allow us to analyze the genetic structure of a population and identify signs of selection. The present study searched for ROH regions in the Faverolle chicken breed. DNA samples from modern individuals and museum Faverolle specimens were obtained and sent for whole-genome sequencing (WGS) with 30× coverage. The results were aligned to the reference genome and subjected to additional filtering. ROH segments were then analyzed using PLINK 1.9. As a result, 10 regions on GGA1, 2, 3, 4, and 13 were identified. A total of 19 genes associated with fat deposition and lipid metabolism (*GBE1*, *CACNA2D1*, *STON1*, *PPP1R21*, *RPL21L1*, *ATP6V0E1*, *CREBRF*, *NKX2-2*, *COMMD1*), fertility (*LHCGR*, *GTF2A1L*, *SAMD5*), muscle development and body weight (*VGLL3*, *CACNA2D1*, *FOXN2*, *ERGIC1*, *RPL26L1*), the shape and relative size of the skeleton (*FAT4*), and autophagy and apoptosis (*BNIP1*) were found. Developmental protein genes (*PAX1*, *NKX2-2*, *NKX2-4*, *NKX2-5*) formed a separate cluster. Probably, selection for the preservation of high flavor characteristics contributed to the consolidation of these ROH regions. The present research enhances our knowledge on the Faverolle breed’s genome and pinpoints their ROH segments that are also specific «selection traces».

## Simple Summary

The present study aimed to analyze the genetic architecture of the Faverolle breed, particular the runs of homozygosity, and to search for traces of selection related to meat productivity. A total of 10 ROH regions on chromosomes 1, 2, 3, 4, and 13 were identified, resulting in 19 genes involved in fat deposition and lipid metabolism, fertility, muscle development and body weight, the shape and relative size of the skeleton (*FAT4*), and autophagy and apoptosis (*BNIP1*) being found. The obtained results allow us to draw a conclusion about regions in the genome subjected to positive selection in the breed and to establish their relationship with meat productivity traits of the breed.

## 1. Introduction

The establishment of the Faverolle breed, which took place on the territory of the modern commune Faverolles in northern France, dates back to the middle of the XIX century [1]. Initially, the breed was classified as a representative of dual-purpose productivity, providing both excellent meat qualities and good egg production in winter. Historically, there are two alternative versions explaining the origin of Faverolles. According to the first one, the Faverolle was bred mainly on the basis of the Silver Gray Dorking and Houdan breeds, with the involvement of some Asian breeds: Brahmas, Langshans, and Cochins [2]. According to the second version, Faverolles were established by crosses between French local five-toed hens, similar to the Dorking breed, with the Maline—an old Belgian meat chicken breed [1].

Modern breed features of Faverolles include polydactyly (five toes), lightly feathered legs, and the presence of decorative feathers on the sides and bottom of the beak, called beards and muffs [3]. A variety of plumage colorations are observed—the most common are salmon, blue, and white, while the least common are black, pale, and ermine colorations [1,3]. To date, Faverolle is classified as a representative of meat productivity breeds. This is primarily due to the fact that the global industry uses specialized egg crosses with high laying performance, which are selected for at least 30 indicators, including egg production, egg weight, feed conversion ratio, shell thickness and strength, etc. [4]. Given long-term selection, at present, none of the local breeds can compete with specialized crosses in terms of egg-laying efficiency [5]; therefore, the selection of Faverolles for increased egg production is economically inappropriate. As poultry meat production has increased, the emphasis on maximizing meat yield [6] has led to industrial broilers now being far more economically viable than native chicken breeds. However, the intensity of selection applied to broilers has altered their physiological homeostasis [7], resulting in increased incidences of muscle abnormalities [8]. These include «wooden breast» myopathy, which is manifested by visual hardness of the pectoral muscles and the presence of out-bulging and pale areas [9], as well as «white striping» myopathy with white striations parallel to muscle fibers [10]. The presence of such abnormalities not only spoils the appearance of meat but also significantly reduces its technological attributes and quality, which affect flavor, aroma, and texture [11]. For example, the presence of myopathies reliably affects the amino acid composition of meat [12]. Hence, although specialized broiler crosses are capable of providing the required amount of meat for the world population [6], there is a growing consumer demand for meat that has a unique flavor, aroma, and texture [13]. This explains the growing interest in meat breeds such as the Faverolle, where the genetic traits for unrivaled meat quality have been preserved for more than 2 centuries.

Meat table qualities combine flavor and aroma [14], although appearance and texture characteristics are occasionally added to them [13]. Meat quality is influenced by many lifetime and postmortem factors: breed, sex, age of slaughter, housing type, diet, pH and postmortem maturation of meat, method and duration of cooking, etc. [14,15,16]. Breed and age of slaughter are considered the main factors [17], although most of the chemical compounds that determine the final meat flavor, including sugars, organic acids, peptides, free amino acids, etc., are formed during postmortem maturation [18]. Intramuscular fat is also worth mentioning, as existing evidence suggests its influence on meat tenderness and juiciness in poultry [19] and other species. Amino acid composition is the most commonly used method to assess meat flavor and aroma [20]; however, it involves slaughtering the individual [21], leading to its exclusion from the breeding process. Thus, whole-genome analysis may be a good solution to this problem, as flavor and aroma characteristics depend on both paratypical and genetic factors [14]. Whole-genome analysis allows us to read the genome of an individual, which provides important data about the genome and genotype–phenotype associations during a lifetime [22]. This, in turn, allows us not only to draw conclusions about the breeding value of the animal but also to use it successfully in selection programs for breed improvement. In addition, whole-genome analysis of different-origin populations can provide valuable data on changes in their genetic structure, including the appearance of runs of homozygosity (ROHs) [22]. ROH regions tend to accumulate in populations as a result of ongoing selection and thus can potentially carry genes responsible for population-specific traits, ranging from unique features, i.e., cold adaptation [23], to productive traits.

Taking into account the abovementioned information, the actual task for effective selection and genetic improvement is the establishment of genetic bases of high flavor meat qualities. The aim of the present study was to establish the genetic basis for high meat quality in the Faverolle breed, based on the identification and study of its ROH regions.

## 2. Materials and Methods

### 2.1. Animal Sampling and DNA Extraction

**Figure 1:**
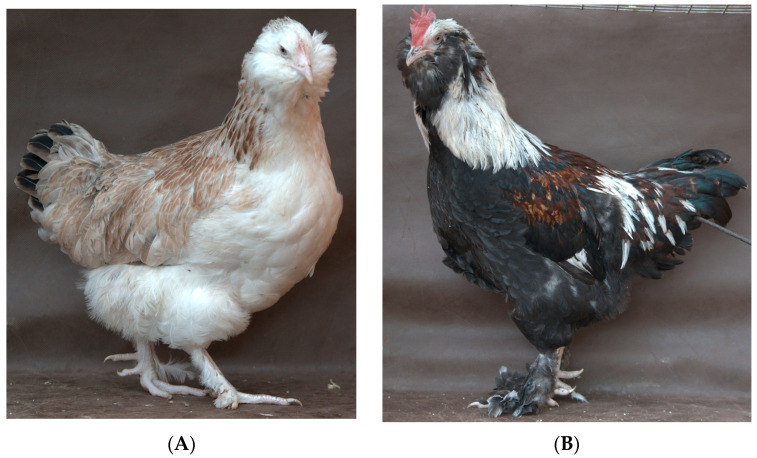
Female (**A**) and male (**B**) specimens of Faverolle breed kept in CCU «Genetic Collection of Rare and Endangered Chicken Breeds» (Pushkin, Saint-Petersburg, Russia).

The Faverolle breed (n = 21) (Figure 1), kept in the Center of Collective Use (CCU) «Genetic Collection of Rare and Endangered Chicken Breeds» (Pushkin, Saint-Petersburg, Russia), as well as stuffed Faverolle specimens (n = 2) from the 1920s, sourced from the National Darwin Museum (Russian Federation, Moscow), were used as material for this study. Blood samples for DNA extraction were collected at the age of 52 weeks from modern Faverolle specimens, while feather samples were obtained from museum specimens. Healthy and non-related Faverolle hens were selected for the analysis. Museum specimens were also female and originally obtained by the museum from different geographic locations. DNA extraction both from blood and feathers was carried out by the standard phenol extraction protocol. Feather DNA extraction involved initially chopping the material and then freezing it for around 24 h. After thawing, the standard phenol protocol was employed for the DNA extraction. The purity and concentration of DNA samples were determined by spectrophotometry on a NanoDrop 2000c (Thermofisher Scientific Inc., Waltham, MA, USA).

### 2.2. Whole-Genome Sequencing

All samples passed quality control and were further sent for whole-genome sequencing with 30× coverage. Libraries were prepared using the library preparation kit TruSeq DNA Nano (Illumina Inc., San-Diego, CA, USA). Sequencing was performed using a NovaSeq 6000 (Illumina Inc., San-Diego, CA, USA), with a read length of 2 × 151 b.p. Quality control showed that 91.46% of reads corresponded to the Q30 level (probability of 1 incorrect base per 1000 reads). Demultiplexing was performed using bcl2fastq2 v2.20 (Illumina Inc., San-Diego, CA, USA), and removal of adapter sequences was performed using Skewer v0.2.2 [24]. Quality assessment and read filtering were performed using FastQ program v 0.12.0. The obtained reads were aligned to the reference genome of Red Jungle Fowl Gallus_gallus_gca000002315v5.GRCg6a from the ENSEMBL international database using bwa-mem2 [25]. As a result, a total of 15,386,681 SNPs were detected.

### 2.3. Data Filtering

SNP filtering was performed using PLINK 1.9 with the following parameters: --maf 0.05, --geno 0.02, and --hwa 0.0001, leaving 10,593,367 SNPs in the analysis. SNPs on sex chromosomes were eliminated from the analysis to exclude the influence of gender on the assessment.

### 2.4. Runs of Homozygosity

ROH regions were analyzed using PLINK 1.9 software and the detectRuns package in the R programming environment, namely RStudio v 2023.12.1+402, using the following parameters: window size, 150 SNPs; window overlap threshold, 0.1; minimum number of SNPs in the region, 200; maximum number of heterozygous SNPs in the window, 1. Borderlines corresponding to the GRCg6a reference genome assembly were then defined for the detected ROH regions. In order to reveal promising candidate genes, the annotation of genes located within these regions was performed in the Ensembl genomic browser.

## 3. Results

**Figure 2:**
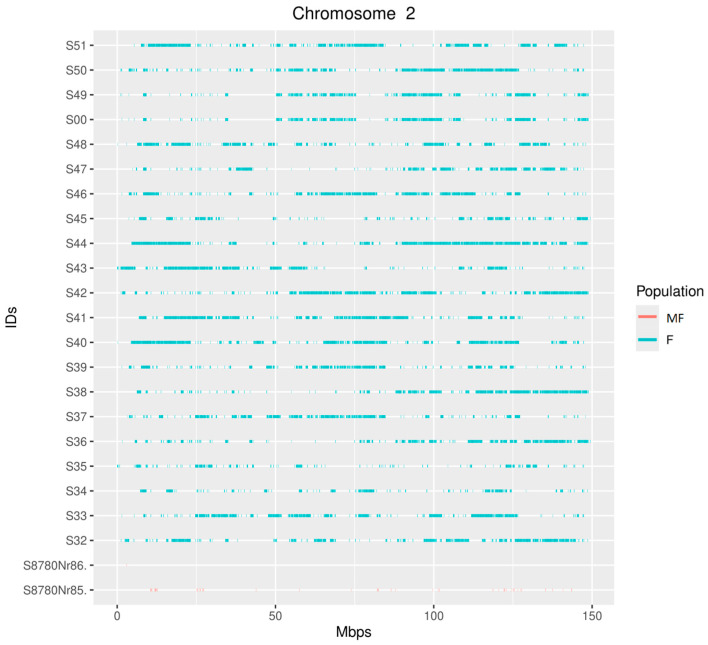
Distribution of runs of homozygosity (ROH) on GGA2 per individual. Line colors indicate the affiliation to chicken population (MF—museum Faverolle specimens; F—modern Faverolle specimens).

**Figure 3:**
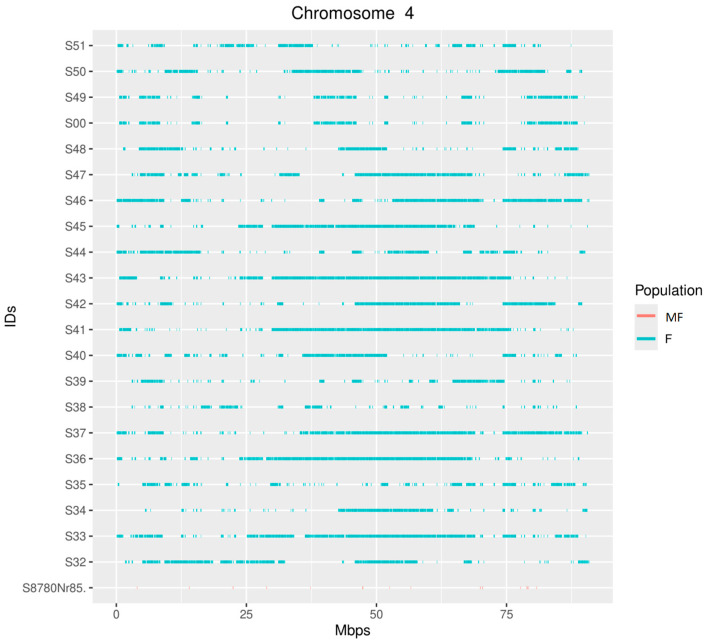
Distribution of runs of homozygosity (ROH) on GGA4 per individual. Line colors indicate the affiliation to chicken population (MF—museum Faverolle specimens; F—modern Faverolle specimens).

Whole-genome sequencing identified 10 ROH regions with a 100% occurrence rate on chromosomes 1, 2, 3, 4, and 13 (Table 1). In the museum samples, only two ROH regions on chromosomes 2 and 4 were detected, with the regions partially overlapping with the modern populations rather than overlapping completely (Figure 2 and Figure 3). These results are probably caused by severe DNA degradation rather than population differences due to selection pressure. The short length of the ROH regions of the museum sample S8780Nr86 makes them difficult to display in the plots, so we added a list of the identified ROH regions in the Supplementary Materials (Table S1).

The highest number of identified ROH regions was found on chromosome GGA3 and amounted to four regions (Figure S1). Thus, the *PAX1*, *NKX2-2*, *NKX2-4*, and *XRN2* genes were localized within the 3451205-3587652 bp region; the *LHCGR*, *GTF2A1L*, and *STON1* genes were localized within 8224869-8317160 bp; and the *FOXN2*, *PPP1R21*, and *COMMD1* genes were localized within 9487340-9599796 bp.

The lowest number of genes was obtained for the 47328186-47481032 bp region, where the *SAMD5* gene is located. The highest number of genes in the detected ROH region was obtained for GGA13 (Figure S2). Thus, the single ROH region on GGA13 contained the *ERGIC1*, *RPL26L1*, *ATP6V0E1*, *CREBRF*, *BNIP1*, and *NKX2-5* genes. A single ROH region containing the *FAT4* gene was also identified on GGA4. Both GGA1 and GGA2 contained two regions each. Regions on GGA1 contained the *VGLL3* and *GBE1* genes, respectively. Finally, the *CACNA2D1* and *PHF14* genes were identified in regions on GGA2 (Figure S3).

## 4. Discussion

Runs of homozygosity are continuous homozygous segments found in human and animal genomes [26]. Factors affecting the size, distribution, and frequency of ROH segments include natural and artificial selection, recombination, linkage disequilibrium, mutation rate, and inbreeding level [27]. ROH regions can be used to infer the historical development of the population [28], since two identical haplotypes were most likely inherited from a common ancestor [29]. Thus, the present study found that modern Faverolle specimens and museum samples share some overlapping ROH regions on GGA 2 and 4, possibly indicating a shared ancestry. Short ROH segments indicate an older common ancestor in the lineage, whereas long regions may be caused by both relatively recent parental inbreeding [30] and selection pressure [31]. ROH studies allow us to analyze the genetic structure of a population and identify signs of natural and artificial selection [32]. Genomic regions under positive selection show increased levels of homozygous sites, which is related to a local decrease in haplotype diversity [31]. Thus, it can be concluded that a fraction of the ROH regions is the actual selection target, which remains stored within these regions [27]. Accordingly, ROH segments are important genome elements that may contain candidate genes responsible for the manifestation of breed-specific, economically important, and other population traits [33].

The present study searched for runs of homozygosity in the Faverolle breed. Thus, two ROH regions with *VGLL3* and *GBE1* genes were identified on GGA1, respectively. *VGLL3* belongs to the mammalian *VGLL1-VGLL4* gene family, which encode transcription co-factors that bind TEAD family transcription factors [34]. *VGLL* genes are quite conserved and have been identified in a number of species including humans, mice, zebrafish, and chickens [35]. First, *VGLL3* was associated with a myogenic cell line during the early embryonic development of mice [36], and then *VGLL2* gene expression was detected in all myogenesis parts (head, torso, and limbs) of chick embryos [35]. The accumulation of an ROH region within *VGLL3* gene in Faverolles may be related to the development of the myogenic cell population during embryonic and postembryonic development. The second gene on GGA1, *GBE1*, encodes an enzyme involved in glycogen biosynthesis, and has been repeatedly associated with the fat deposition process in chickens. A recent study found an association of *GBE1* with intramuscular fat deposition in pectoral muscle [37]. The same study found that carbohydrate metabolism plays an important role in intramuscular fat deposition, while fatty acid and glycerol metabolism regulate abdominal fat deposition. Another study observed that *GBE1* expression levels in liver tissues were significantly higher in broilers with high fat deposition [38]. This is explained by the fact that in avian species, the liver is primarily responsible for lipogenesis, whereas adipocytes serve as triglyceride depots [39]. Lastly, a comparative study found that *GBE1* expression level was higher in fast-growing chickens compared to slow-growing chickens [40]. Given that fat deposits contribute significantly to the juiciness, flavor, aroma, and other organoleptic properties of meat [40], it can be concluded that the presence of the ROH region in the *GBE1* gene is a trace of selection of Faverolles for high meat qualities.

The ROH region with the *CACNA2D1* is of increased interest on GGA2. Transcriptome analysis identified *CACNA2D1* as a promising candidate gene associated with carcass weight and intramuscular fat deposition in cattle [41]. Moreover, this gene showed increased expression in *Musculus longissimus lumborum*, which supports its potential influence on marbling quality in different tissues [41]. These findings are consistent with the present study and explain the establishment of the ROH region within *CACNA2D1*, since selection in Faverolles was aimed at improving meat qualities and flavor characteristics.

A region with the *PHF14* gene encoding a histone-binding protein has been identified on GGA2. *PHF14* plays an important role in lung development and function, as polymorphisms in this gene have been associated with lizard tolerance to high-altitude hypoxia [42], and the depletion of *PHF14* led to respiratory failure and death in mice [43]. A partial overlap with the museum specimens was found in this region, which suggests that the fixation of this region is due to more ancient inbreeding. The DNA of the museum samples is highly degraded, making it difficult to assess the true ROH length, but the detection of this fragment suggests that ROH segments on GGA2 are likely to be regions with traces of “ancient” inbreeding.

Four ROH regions were identified for GGA3 (Table 1). Among them, the region with the *LHCGR*, *GTF2A1L*, and *STON1* genes is of particular interest. *LHCGR* is known to encode a receptor for luteinizing hormone and choriogonadotropin. Its involvement in normal puberty and fertility in both males and females has been noted. *LHCGR* polymorphisms are associated with polycystic ovary syndrome in women with a high body mass index (BMI) and waist-to-hip ratio [44]. A comparative analysis of Lohmann laying hens and Liangshan Yanying hens revealed that high *LHCGR* expression levels in the ovaries of Lohmann laying hens were associated with high reproductive function [45]. *GTF2A1L* presumably encodes a specific testis transcription factor. Abnormal expression of this gene was previously proposed as one of the causes of infertility [46]. The last gene in the region, *STON1*, encodes a component of the endocytic machinery and was previously associated with adipocyte metabolism [47]. The clustering of genes associated with fat deposition and fertility is consistent with the existing relationship between lipid metabolism and fertility in humans, animals, and plants [48,49]. It can be assumed that this ROH region is formed by the preferential conservation of *STON1*, and other genes remained in a homozygous state due to their proximity to this gene and close interaction with it.

A relationship with qualitative meat productivity traits can also be assumed for the region with the *FOXN2*, *PPP1R21*, and *COMMD1* genes. A comparative GWAS study indicated that the *FOXN2* gene, which encodes a transcription factor, is associated with live birth weight in ducks [50]. *PPP1R21* acts as a cofactor of protein phosphatase 1 (PP1), which is required for cell division, the control of glycogen metabolism, protein synthesis, and muscle contractility. Polymorphisms in *PPP1R21* have been previously associated with neurodegenerative diseases with numerous symptoms, including muscle weakness [51]. The last gene of the region, *COMMD1*, encodes a pleiotropic factor involved in the regulation of many cellular and physiological processes, including cholesterol homeostasis, oxidative stress, DNA damage response, etc. [52]. Studies have noted that *COMMD1* plays a crucial role in the regulation of the NF-κB signaling pathway, which is vital for immune responses and the regulation of inflammation [53]. Hence, genes of this ROH region are associated with lipid metabolism and oxidative stress. Oxidative stress, depending on the intensity and duration of its influence, can both positively and negatively affect adipogenesis [54], which, in turn, is crucial for body metabolic homeostasis [55]. Understanding the influence of these genes on Faverolle meat traits requires additional studies focused on molecular interactions.

The next ROH region on GGA3 contained the *PAX1*, *NKX2-2*, *NKX2-4*, and *XRN2* genes. This group encodes activators and transcription factors, and all genes except *XRN2* belong to the developmental proteins group. Developmental proteins are highly conserved genes that act early in embryogenesis and ensure its proper course. Consistent with its biological function, mutations in the *PAX1* gene are associated with malformations of the vertebral column in avian species [56]. Besides involvement in the regulation of thymus epithelial precursor development [57], *PAX1* is also able to inhibit the WNT signaling pathway in vertebrate cells [58]. *NKX2-2* has been suggested to be involved in central nervous system morphogenesis [59]. A mutation in this gene resulted in severe neonatal diabetes, obesity, and developmental delay [60]. The last gene, *XRN2*, encodes an exoribonuclease and plays an important role in DNA damage response. Loss of *XRN2* leads to replication stress, DNA double-strand breaks, hypersensitivity of cells to DNA-damaging agents and, consequently, to genomic instability [61]. The formation of this ROH region is largely due to the importance of genes for the normal course of embryogenesis. The involvement of these genes in the endocrine system suggests that the fixation of this region is related to its influence on metabolism. However, further research is needed to identify specific causal interactions.

The last region on GGA3 is located in the *SAMD5* gene. Data on its biological function are scarce, but there are a number of studies in which it has been associated with fertility parameters. Existing studies have proposed *SAMD5* as a candidate gene associated with fertility in female pigs [62] and male cattle [63], and an association with sperm neck damage was also found in frozen–thawed semen of Holstein bulls [64].

A single ROH region with the *FAT4* gene was identified on GGA4. Partial overlap in this region was detected for museum specimens, which suggests that it was under positive selection. The FAT4 gene encodes a member of the cadherin family involved in cell adhesion processes. Mutations in it are known to affect osteoblast differentiation [65]. A study in mice revealed that *FAT4*, together with *DCHS1*, controls cell orientation during early skeletal condensation, determining its shape and relative size [66]. In terms of meat productivity, *FAT4* could form an ROH region due to the fact that skeleton shape and relative size, together with muscles, form the musculoskeletal system, which determines the genetic potential of an individual for growth [67,68].

Finally, the *ERGIC1*, *RPL26L1*, *ATP6V0E1*, *CREBRF*, *BNIP1*, and *NKX2-5* genes were identified in the single ROH region on GGA13. *ERGIC1* encodes a membrane protein presumably involved in transport between the endoplasmic reticulum (ER) and the Golgi. Interestingly, rs43350563 in the *ERGIC1* gene had a significant effect on body weight in beef cattle of Simmental and Red Angus breeds [69]. Also, *ERGIC1* is a candidate gene associated with arthrogryposis, a systemic disease of the musculoskeletal system characterized by contracture and deformity of the limbs and underdevelopment of joints and muscles [70]. *RPL26L1*, a paralog of which is a member of the RPL26 ribosomal protein family, can be included among the genes potentially affecting meat productivity. Cells lacking *RPL26* and *RPL26L* exhibit severe growth defects [71]. *ATP6V0E1* encodes an enzyme involved in ion transport. The effect of this gene on adipose tissue, obesity, and skeletal muscle development in humans and mice has been previously observed [72]. A GWAS study conducted on Hereford and Angus beef cattle associated *ATP6V0E1* with mid-test metabolic weight [73]. Moreover, *ATP6V0E1* was identified as one of the key hub genes positively associated with glycolipid biosynthesis in Nelore cattle [74]. The *CREBRF* gene, involved in stress response, transcription, and its regulation, was previously identified as a key regulator of muscle energy metabolism [75], while variants in this gene were found to affect BMI in Samoans, a unique population with high prevalence of obesity [76]. A study in pigs found that a variant of *CREBRF* is characterized by a dramatic increase in subcutaneous fat deposition [77]. The *BNIP1* gene may have an indirect effect on meat productivity. *BNIP1*-deficient cells exhibit a defect in the fusion of autophagosomes and lysosomes, which disrupts the autophagy process essential for skeletal development [78]. Finally, *NKX2-5*, which encodes a homeobox-containing transcription factor, was also identified in the region. This gene is known to play an important role in cardiac development and regeneration, as evidenced by mutations leading to various cardiac malformations [79]. Thus, all ROH region genes on GGA13, besides *NKX2-5*, are associated with the musculoskeletal system and are involved in the metabolic processes of the body, ranging from glycolipid biosynthesis and lipid metabolism to energy metabolism in muscle.

## 5. Conclusions

Whole-genome sequencing identified 10 ROH regions on GGA1, 2, 3, 4, and 13. Partial overlap with museum specimens was found for regions on GGA2 and GGA4, which suggests that consolidation of these ROH regions in Faverolles occurred quite a long time ago. This also suggests that these regions may contain genes important for meat production, which historically have been the basis for selection in the Faverolle breed. The highest number of regions was detected on GGA3 and amounted to four regions, whereas GGA1 and 2 had two regions each. The lowest number of regions was found on GGA4 and 13 and amounted to one region each. A total of 19 genes associated with fat deposition and lipid metabolism (*GBE1*, *CACNA2D1*, *STON1*, *PPP1R21*, *RPL21L1*, *ATP6V0E1*, *CREBRF*, *NKX2-2*, *COMMD1*), fertility (*LHCGR*, *GTF2A1L*, *SAMD5*), muscle development and body weight (*VGLL3*, *CACNA2D1*, *FOXN2*, *ERGIC1*, *RPL26L1*), the shape and relative size of the skeleton (*FAT4*), and autophagy and apoptosis (*BNIP1*) were found. In addition to the above, developmental protein genes (*PAX1*, *NKX2-2*, *NKX2-4*, *NKX2-5*) formed a separate cluster. The identification of regions associated with lipid metabolism and fertility is consistent with the existing relationship between them and further confirms that these traits are interdependent. The presence of a large cluster of lipid metabolism genes in Faverolles can be explained by the fact that fats determine such meat characteristics as tenderness. Probably, selection for the preservation of high flavor characteristics contributed to the consolidation of this genotype variation. A number of genes, including developmental protein genes, are involved in the development of the musculoskeletal system. To understand the significance of these genes in Faverolles, it should be noted that the genetic potential of an individual to grow is limited by the shape and size of the musculoskeletal system. Perhaps the formation of ROH regions with these genes is also due to selection performed to consolidate both the direction of productivity and unique flavor characteristics of meat. The present research enhances our knowledge of the Faverolle breed’s genome and pinpoints its ROH regions that are also specific «selection traces».

## Figures and Tables

**Table 1:** List of ROH regions and genes identified in them in Faverolle breed.

GGA ^1^	Region	Gene	Modern Specimens	Museum Specimens
1	94,324,548–94,452,368	*VGLL3*	+	-
	96,486,174–96,785,153	*GBE1*	+	-
2	10,335,960–10,664,230	*CACNA2D1*	+	-
	26,156,990–26,542,990	*PHF14*	+	Partial overlap
3	47,328,186–47,481,032	*SAMD5*	+	-
	3,451,205–3,587,652	*PAX1*, *NKX2-2*, *NKX2-4*, *XRN2*	+	-
	8,224,869–8,317,160	*LHCGR*, *GTF2A1L*, *STON*	+	-
	9,487,340–9,599,796	*FOXN2*, *PPP1R21*, *COMMD1*	+	-
4	52,155,823–52,596,893	*FAT4*	+	Partial Overlap ^2^
13	9,814,823–9,936,123	*ERGIC1*, *RPL26L1*, *ATP6V0E1*, *CREBRF*, *BNIP1*, *NKX2-5*	+	-

^1^ Gallus Gallus chromosome; ^2^ identified only for museum Faverolle sample S8780Nr85.

## Data Availability

Data will be made accessible from corresponding authors upon reasonable request.

## Supplementary Materials

The following supporting information can be downloaded at: https://www.mdpi.com/article/10.3390/ani15101487/s1, Figure S1: Distribution of runs of homozygosity (ROH) on GGA3 per individual. Line colors indicate the affiliation to chicken population (MF–museum Faverolle specimens, F–modern Faverolle specimens); Figure S2: Distribution of runs of homozygosity (ROH) on GGA13 per individual. Line colors indicate the affiliation to chicken population (MF-museum Faverolle specimens, F–modern Faverolle specimens); Figure S3: Distribution of runs of homozygosity (ROH) on GGA1 per individual. Line colors indicate the affiliation to chicken population (MF-museum Faverolle specimens, F–modern Faverolle specimens). Table S1: List of all ROH regions identified in the museum Faverolle specimens.
