# Supplementary material for: Search for Ancient Selection Traces in Faverolle Chicken Breed (Gallus gallus domesticus) Based on Runs of Homozygosity Analysis
Source: Animals (Basel). 2025 May 20;15(10):1487. doi: 10.3390/ani15101487 (PMC12108394; doi:10.3390/ani15101487)
Supplement: Supplementary file 1 [file animals-15-01487-s001.zip › Supplementary Figures_v3.pdf]

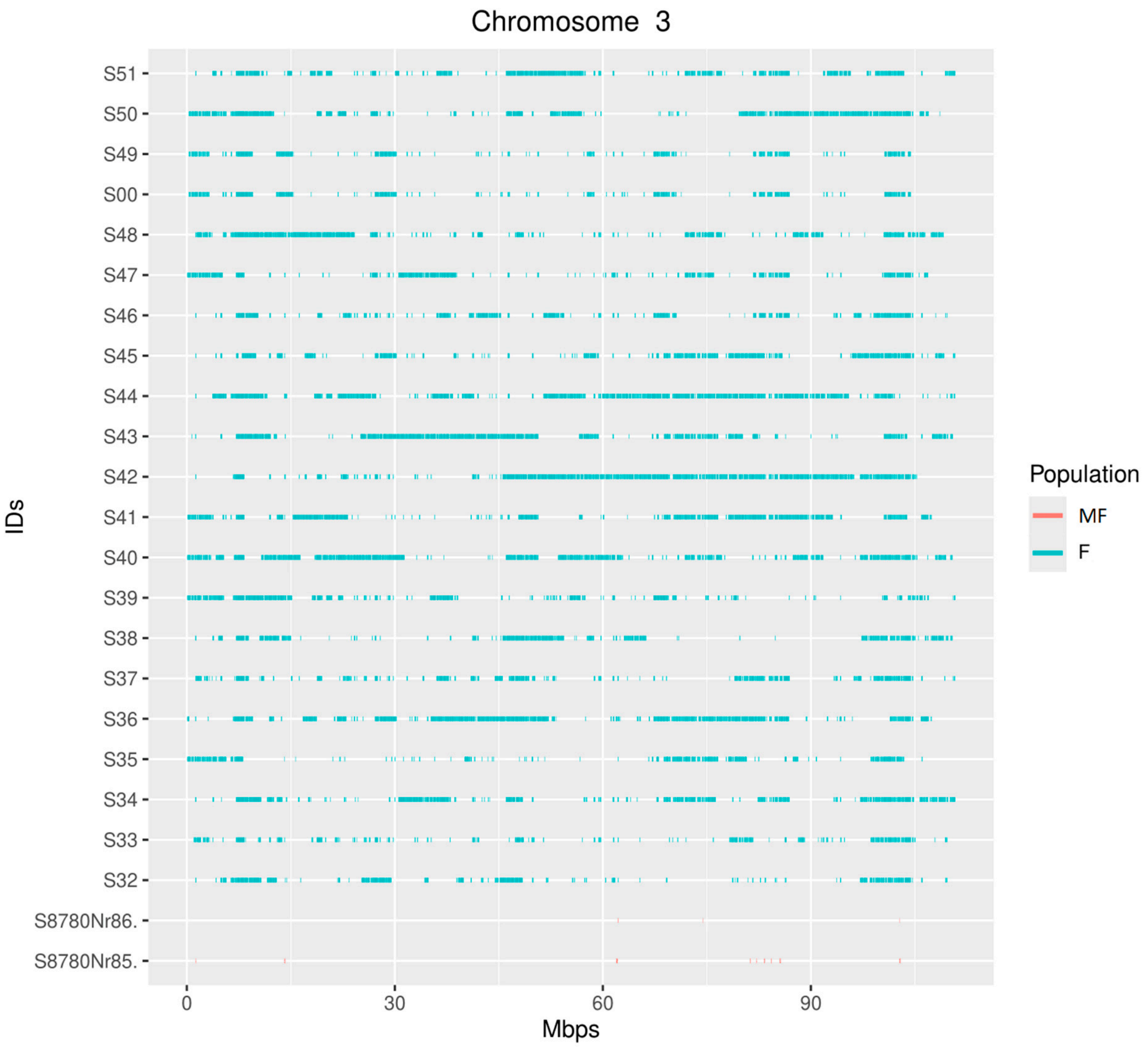

Figure S1. Distribution of runs of homozygosity (ROH) on GGA3 per individual. Line colors indicate the affiliation to chicken population (MF – museum Faverolle specimens, F – modern Faverolle specimens).

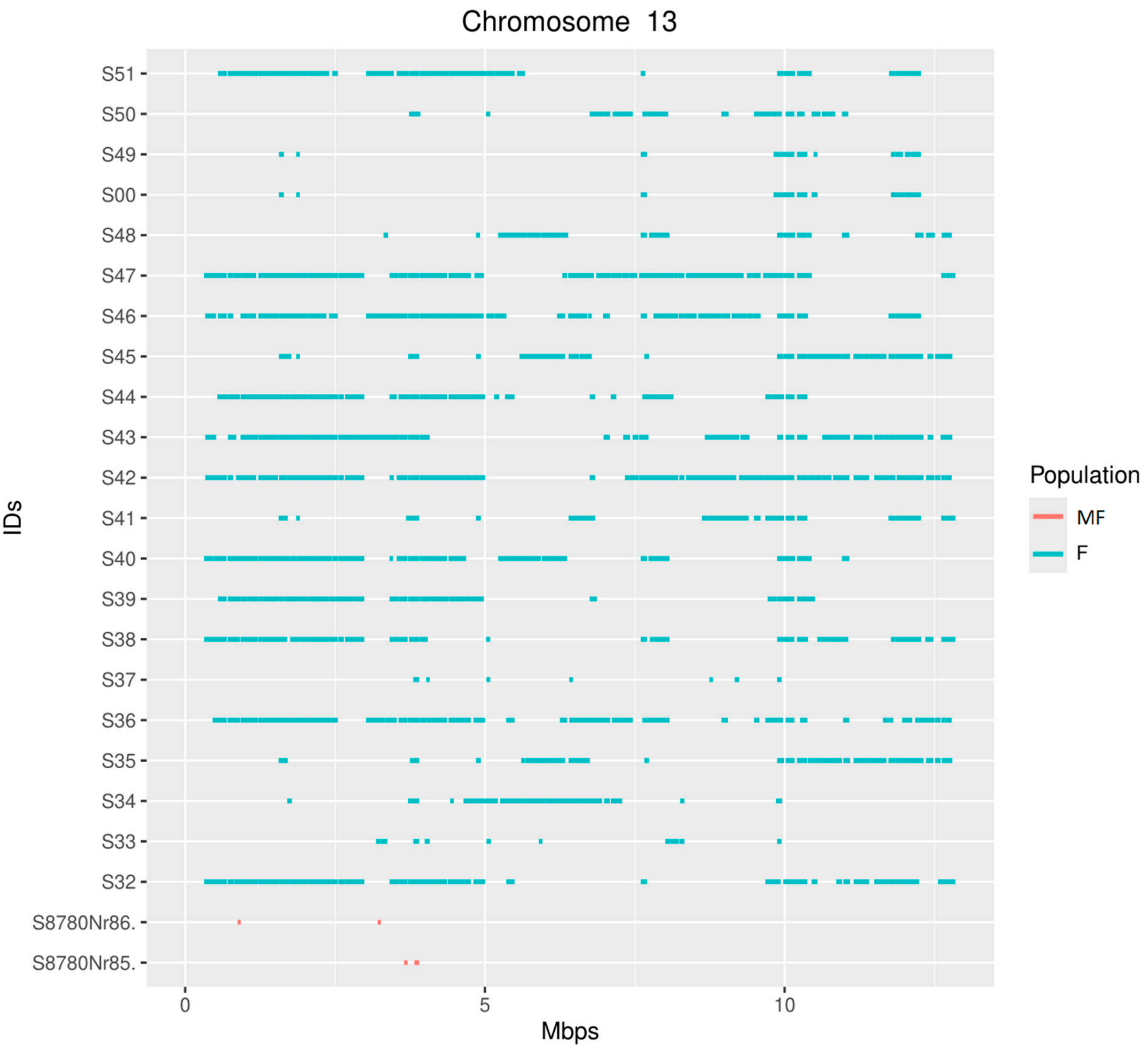

Figure S2. Distribution of runs of homozygosity (ROH) on GGA13 per individual. Line colors indicate the affiliation to chicken population (MF - museum Faverolle specimens, F – modern Faverolle specimens).

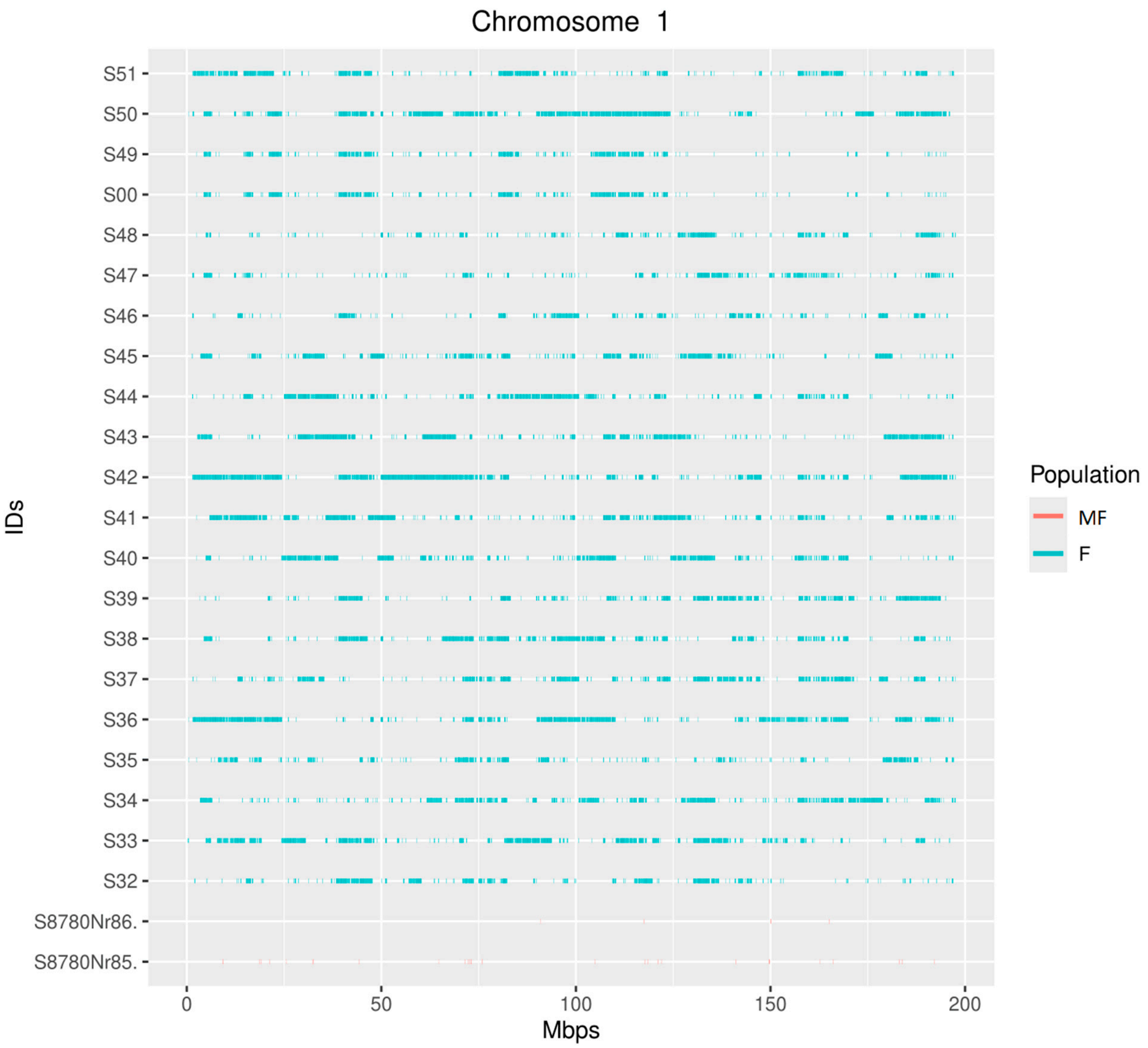

Figure S3. Distribution of runs of homozygosity (ROH) on GGA1 per individual. Line colors indicate the affiliation to chicken population (MF - museum Faverolle specimens, F – modern Faverolle specimens).
